# Laser-Induced Synthesis of Composite Materials Based on Iridium, Gold and Platinum for Non-Enzymatic Glucose Sensing

**DOI:** 10.3390/ma13153359

**Published:** 2020-07-29

**Authors:** Maxim S. Panov, Evgeniia M. Khairullina, Filipp S. Vshivtcev, Mikhail N. Ryazantsev, Ilya I. Tumkin

**Affiliations:** 1 Saint Petersburg State University, 7/9 Universitetskaya Nab., 199034 St. Petersburg, Russia; m.s.panov@spbu.ru (M.S.P.); e.khayrullina@spbu.ru (E.M.K.); 2 Nanotechnology Research and Education Centre of the Russian Academy of Sciences, Saint Petersburg Academic University, 194021 St. Petersburg, Russia; vforvshivcev@gmail.com

**Keywords:** laser-induced deposition of metal microstructures, iridium, gold, platinum, composites, enzyme-free sensing, glucose

## Abstract

A simple approach for in situ laser-induced modification of iridium-based materials to increase their electrocatalytic activity towards enzyme-free glucose sensing was proposed. For this purpose, we deposited gold and platinum separately and as a mixture on the surface of pre-synthesized iridium microstructures upon laser irradiation at a wavelength of 532 nm. Then, we carried out the comparative investigation of their morphology, elemental and phase composition as well as their electrochemical properties. The best morphology and, as a result, the highest sensitivity (~9960 µA/mM cm^2^) with respect to non-enzymatic determination of D-glucose were demonstrated by iridium-gold-platinum microstructures also showing low limit of detection (~0.12 µM), a wide linear range (0.5 µM–1 mM) along with good selectivity, reproducibility and stability.

## 1. Introduction

There are many methods for determination of biologically significant compounds such as hydrogen peroxide, glucose, amino acids and many others [1,2]. Methods based on usage of the electrochemical sensors are the most useful and effective among them [3,4]. Electrochemical sensors are devices designed for qualitative and quantitative analysis of liquid and gaseous media, in which the analytical signal is provided by the flow of electrons emerging due to an electrochemical reaction in the near-electrode space. The central feature of any sensors that distinguishes them from other analytical devices is ability to conduct field analysis in real time and minimal sample preparation. Electrochemical sensing and monitoring of glucose as one of the most important substrates for the diagnosis and treatment of diabetes attract particular attention in this regard [4]. The main principle of operation of the most well-known glucose meters and sensors is an electrochemical method for measuring glucose concentration based on its enzymatic cleavage. Such measurements proceed through two stages. At the first stage, glucose from the blood ends up in the test area and under the action of the enzyme glucose oxidase is broken down into gluconic acid and hydrogen peroxide [3,5,6]. At the second stage, each hydrogen peroxide molecule under the action of a small electric field decays to form an oxygen molecule, two protons and two electrons. Further, each glucose molecule gives off two electrons creating an electric current, which, in turn, is detected by the analyzer. Nevertheless, it is known that these enzyme sensors for glucose detection have a number of disadvantages: low accuracy of detection, low stability due to enzyme decomposition and high sensitivity to pH and humidity of the environment leading to a short service life of such devices [6]. The reason for this is the interfering influence of biologically active substances present in the blood as well as the type of materials used as electrodes in these sensors. Indeed, for example, thin-film electrodes based on gold or platinum with immobilized glucose oxidase are frequently used, in which activity of an enzyme decreases very quickly over time, thereby reducing the lifetime of such sensors. Moreover, the methods used for production of thin-film electrodes (e.g., vacuum deposition) do not provide high reproducibility, so a separate calibration is required for each sensor, which, in turn, affects the accuracy of determining the glucose concentration in the blood [6,7,8,9,10,11,12]. In opposite, our approach proposed avoids such disadvantages and assumes a more selective and accurate invasive biochemical blood analysis that can be performed both in the laboratory and at home. This approach is based on the use of laser-induced metal deposition from solution (LCLD) to produce enzyme-free electrochemical microsensors suitable for glucose detection [13]. In LCLD, the metal reduction reaction occurs in a local volume of a solution within the focus of the laser beam leading to its deposition on the surface of a dielectric substrate (e.g., glass, glass-ceramics etc.). This method allows the synthesis of electrocatalytically active materials based on different metals and has a number of advantages over many known analogues due to its simplicity, low cost and sufficient efficiency [14,15,16,17,18,19,20,21]. The sensors synthesized by LCLD can be applied for direct detection of biologically important analytes. It is known that enzymatic detection of such analytes is complicated by high values of their potentials. In contrast, in non-enzymatic detection, the value of these potentials can be reduced due to highly developed structure of the electrode materials, which can also be modified with gold or platinum micro- or nanoscaled particles resulting in catalysis of ox-red reactions of analytes (e.g., glucose) on the surface of an electrode [22,23,24]. As a result, non-enzymatic sensing combines the advantages of the electrochemical methods and the unique properties of nanomaterials exhibiting large surface area due to three-dimensional structures of such nanoparticles of catalytically active and biologically compatible metals as gold and platinum. This leads to significant decrease of the analysis time and increase of sensitivity, stability and intensity of the analytical signal in the determination of glucose concentration.

In this work, we synthesized mono- and polymetallic microstructures based on iridium, gold and platinum using LCLD technique. We also performed comparative study of their morphology, composition and electrochemical properties. Iridium-containing metal and oxide micro- and nanostructures demonstrated rather good electrocatalytic activity towards pH sensing and enzyme-free detection of many biologically important substrates [25,26,27,28]. This makes them a good choice for application in non-enzymatic glucose sensing.

## 2. Materials and Methods

Analytically graded chloro(triphenylphosphine)gold(I), tris(2-phenylpyridine)iridium(III), dichloro(dicyclopentadienyl)platinum(II), *N*,*N*-dimethylformamide (DMF) were used as received from Sigma Aldrich (St. Louis, MO, USA) without any further purification. All solutions were prepared with deionized water with a resistivity of greater than 18.1 MΩ·cm^−1^. The compositions of solutions for LCLD are shown in Table 1. Silica-based glass-ceramics slides were used as substrate for laser-induced deposition process.

The scheme of the experimental setup and its detailed description can be found in Supplementary Materials (Figure S1) and elsewhere [13]. An neodymium-doped yttrium aluminum garnet (Nd:YAG) diode-pumped solid-state cw laser (Changchun New Industries Optoelectronics Technology Co., Ltd., Changchun, China) with a wavelength of 532 nm was used as a light source for the thermo-induced reduction and deposition of metals. The output from a laser is split into two portions. First portion of the laser output provides in situ monitoring the process of laser-induced synthesis using web-camera. In turn, the second portion is used to govern reduction reaction. Microdeposits were produced by scanning the laser beam focused between the plating solution and dielectric substrate using an XYZ controlled motorized stage. Consequently, we were able to deposit the 8 mm long and 70–90 μm wide iridium (Ir) lines at the laser power of 1500 mW and at the scanning speed of 2.5 μm s^−1^. Finally, the synthesized Ir lines were modified by the consecutive laser-induced deposition of gold and platinum on the top of them using the same deposition rate and at laser power of 1100 mW.

Scanning electron microscopy (SEM) images of the synthesized iridium containing microdeposits were obtained with Zeiss Supra 40 VP scanning electron microscope (Oberkochen, Germany). Energy-dispersive X-ray spectroscopy (EDX) was used to identify atomic composition of microelectrodes, all samples were studied using an INCA X-Act EDX analyzer (Oxford Instruments, Abingdon, UK) coupled with SEM.

XRD measurements were carried out on Bruker D2 Phaser diffractometer equipped with LynxEye detector (Karlsruhe, Germany) using CuKα (0.1542 nm) radiation in the 2θ angle range of 0–100°.

The electronic absorption spectra of the DMF solutions of triphenylphosphine chloride of gold(I), dicyclopentadienyl platinum(II) dichloride and tris(2-phenylpyridine)iridium(III) were recorded using UV-vis spectrophotometer Shimadzu UV-2550 (Duisburg, Germany).

Electrochemical characterization was performed in a conventional three-electrode cell at ambient temperature, using Elins P30I potentiostat (Electrochemical Instruments Ltd., Chernogolovka, Russia). Platinum wire, Ag/AgCl electrode and Ir-based microelectrodes was used as counter, reference and working electrodes respectively. All electrochemical measurements were done in 0.1 M sodium hydroxide saturated with Ar as background solution. The electrocatalytic activity of the fabricated Ir-based materials towards glucose was studied using CV and CA techniques. Cyclic voltammetry (CV) experiments were executed at a scan rate of 50 mV·s^−1^ between −0.8 and 0.8 V vs. Ag/AgCl. CA was implement by the addition of the solutions of D-glucose of different concentrations to background solution with simultaneous stirring.

## 3. Results and Discussion

The microstructures based on iridium, gold and platinum were synthesized using the method of laser-induced deposition of metals from solution (LCLD). Thus, we produced iridium (Ir) microdeposits from a solution of tris(2-phenylpyridine)iridium(III) in *N*,*N*-dimethylformamide (DMF) on the surface of glass-ceramics. Iridium-platinum (Ir-Pt) and iridium-gold (Ir-Au) microstructures were obtained by consecutive laser-induced deposition of platinum and gold from DMF solutions of dicyclopentadienyl platinum(II) dichloride and triphenylphosphine chloride of gold(I), respectively, on the surface of pre-synthesized iridium microdeposits. Iridium-gold-platinum (Ir-Au-Pt) microstructures were produced by consecutive laser-induced deposition of platinum on the surface of the pre-synthesized iridium-gold (Ir-Au).

**Figure 1:**
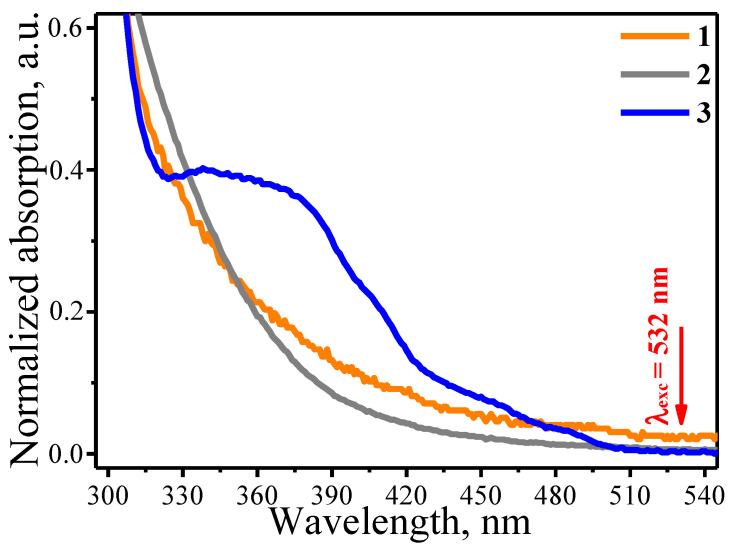
UV-vis absorption spectra normalized to unity at the absorption maximum of the DMF solutions of triphenylphosphine chloride of gold(I) (1), dicyclopentadienyl platinum(II) dichloride (2) and tris(2-phenylpyridine)iridium(III) (3).

In fact, photochemical reactions potentially can contribute in decomposition of metal complexes used for LCLD in this study. Photochemistry of such complexes is defined by the photochemical activity of their excited states associated with the ligand-to-metal charge transfer (LMCT). In general, the electronic absorption spectra of metal complexes reveal intense LMCT bands in the UV-vis region as well as absorption bands located at longer wavelengths (including near-IR range) assigned to d-d transitions. The excitation to the d-d states does not induce the photoreaction, whereas the LMCT excited states are dissociative and lead to the reduction of metal complex followed by formation of metallic structures [29,30,31,32]. In turn, the 532 nm excitation used in the current work does not populate the photochemically active LMCT states of 1, 2 and 3 (Figure 1) meaning that in our case we observe thermo-induced metal reduction process.

**Figure 2:**
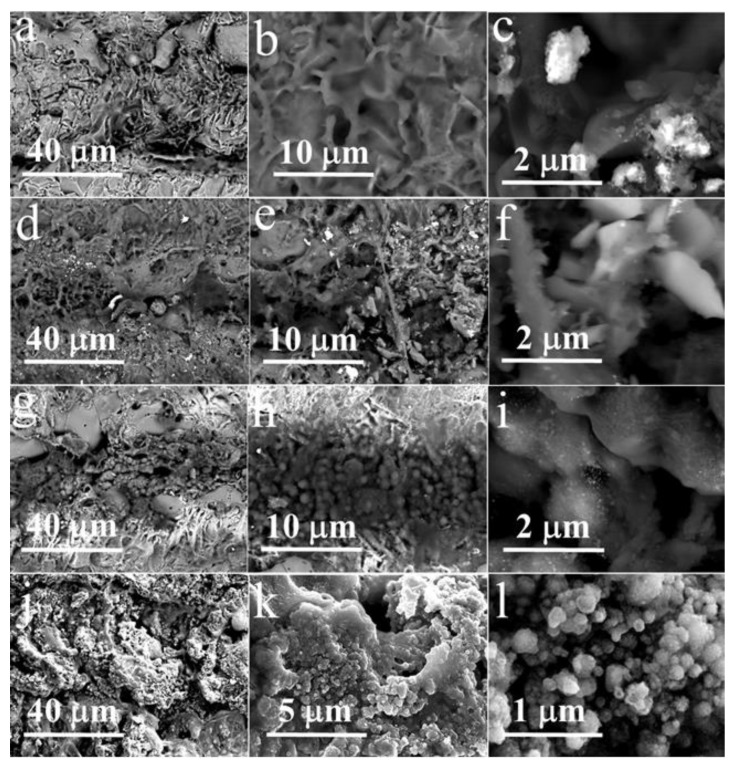
SEM images of (**a**–**c**) iridium (Ir), (**d**–**f**) iridium-platinum (Ir-Pt), (**g**–**i**) iridium-gold (Ir-Au) and (**j**–**l**) iridium-gold-platinum (Ir-Au-Pt) microstructures.

**Figure 3:**
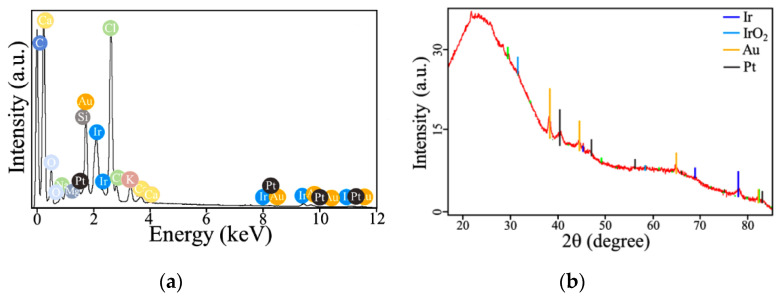
EDX spectrum (**a**) and XRD pattern (**b**) of iridium-gold-platinum (Ir-Au-Pt) microstructures.

Figure 2 demonstrates the morphology of these Ir-based materials examined using scanning electron microscopy (SEM). The surface of the fabricated iridium microdeposits has continuous and uniform structure (Figure 2a–c). Modification of the surface of iridium with platinum did not lead to a significant increase in surface development. SEM revealed that platinum forms micro-sized structures of irregular shape on the surface of iridium (Figure 2d–f). Iridium microdeposits doped with gold have both insular and continuous structure, the surface of which is more developed in comparison with two previous materials (Figure 2g–i). Herein, gold is deposited as separate micro- and nanoscaled drops (Figure 2i), which are prone to agglomeration (Figure 2h). The most interesting results were obtained by successive deposition of platinum on the surface of Ir-Au. Ir-Au-Pt microstructures are similar to Ir-Au in a number of points, but have several significant differences (Figure 2j–l). These microdeposits have highly developed surface consisting of spherical particles with a diameter of 40–400 nm of gold and platinum, both individually and as a mixture (Figure 2l). Unlike Ir-Au, there is no complete agglomeration of small particles into clusters, but there is a significant variation in their size (Figure 2k,l). The results of energy-dispersive X-ray (EDX) and X-ray diffraction (XRD) analysis are shown in Figure 3 and Supplementary Materials (Figures S2–S7). EDX-analysis exhibited the presence of some other elements (besides iridium, gold and platinum) that are most likely associated with the substrate material and components of the plating solutions. XRD of the synthesized microstructures showed the presence of a polyphase multicomponent mixture containing metal and oxide phases (Figure 3). For all materials, the metal phase is iridium, whereas, the oxide phase is iridium dioxide (IrO_2_). Additional metal phases were observed for Ir-Pt-platinum and for Ir-Au-gold. The presence of IrO_2_ in all microdeposits is consistent with their sufficiently high values of electrical resistance (~0.4–1.5 kΩ), which are close to those of semiconductors.

**Figure 4:**
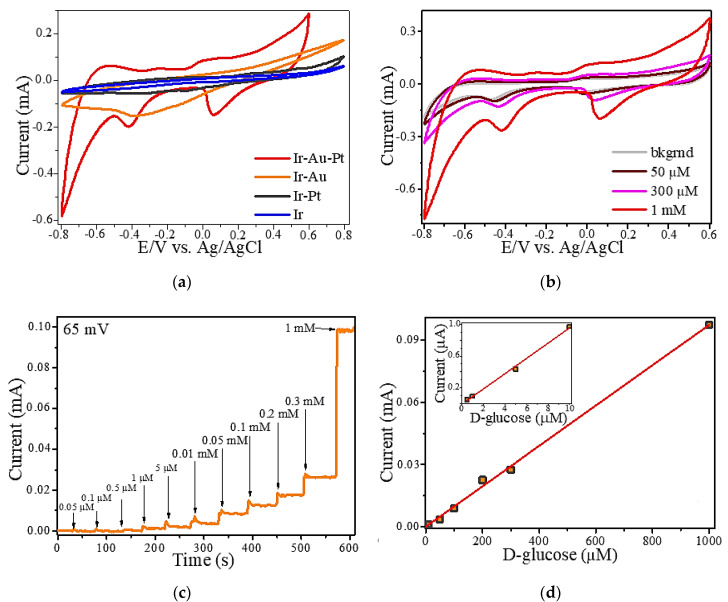
Cyclic voltammograms (CVs) of microstructures based on iridium, gold and platinum recorded in 0.1 M NaOH containing 1 mM D-glucose (**a**). CV of Ir-Au-Pt recorded in 0.1 M NaOH with different concentration of D-glucose (**b**). Amperogram of Ir-Au-Pt obtained in 0.1 M NaOH with different concentration of D-glucose (**c**). Linear dependence of the measured Faraday current on the D-glucose concentrations (**d**).

Figure 4a illustrates cyclic voltammograms (CVs) of all types of iridium-containing microstructures recorded in solutions of 1 mM D-glucose. Here, it should be noted that the area and shape of CV curve of Ir-Au-Pt differs significantly from other Ir-based microelectrodes. It is known that the area of a CV is directly related to the degree of development of the surface of an electrode. Moreover, the value of this parameter determines the sensitivity of an electrode with respect to a particular analyte. As shown in Figure 4a, modification of the iridium (Ir) microdeposits with platinum does not considerably improve its sensory properties. In opposite, modification of iridium with gold and, in particular, with a mixture of gold and platinum causes a noticeable increase in the area of CV curve. As a result, consecutive laser-induced deposition of iridium, gold and platinum allows producing a microelectrode with a much higher analytical response. Considering this, our further electrochemical studies were focused only on Ir-Au-Pt. Figure 4b presents cyclic voltamperograms of Ir-Au-Pt microstructures recorded in a background solution (0.1 M NaOH) with different concentration of D-glucose. The peaks of potentials corresponding to electrocatalytic oxidation and reduction of glucose for Ir-Au-Pt have a pronounced character for the entire region of analyte concentrations. The region of anodic glucose oxidation is located approximately between −100 and 100 mV, moreover, there are two regions of cathodic oxidation of glucose lying between −500 and −300 mV and between 0 and 100 mV. The second region of cathodic glucose oxidation shifts with an increase of the concentration of D-glucose (Figure 4b).

**Figure 5:**
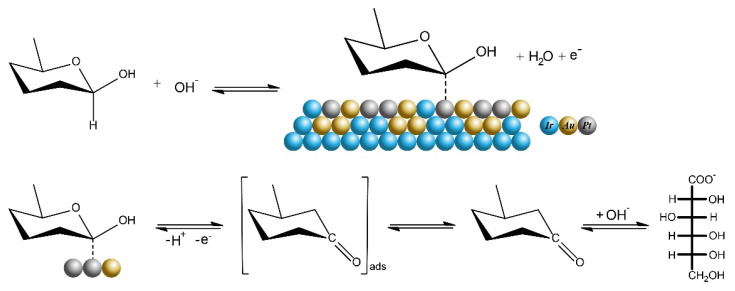
Schematic representation of a possible mechanism for oxidation of glucose at Ir-Au-Pt microelectrode.

In addition, the amperometric method was also used to evaluate the electrocatalytic activity of Ir-Au-Pt microelectrode towards glucose detection. Figure 4c illustrates a typical amperogram that characterizes the reaction of Ir-Au-Pt to successive additions of D-glucose to the background solution at a potential of 65 mV corresponding to the region of the double electric layer [24]. It is clear that an increase of the concentration of D-glucose leads to an increase in the analytical signal. The linear dependence of the Faraday current for Ir-Au-Pt measured at a potential of 65 mV on concentration of D-glucose is shown in Figure 4d. It can be seen that Ir-Au-Pt demonstrates a linear range of enzyme-free glucose determination lying between 0.5 µM and 1 mM. The limit of detection (LOD) of glucose for Ir-Au-Pt microelectrode was calculated as LOD = 3 S/b, where S is the standard deviation from linearity and b is the slope of the calibration curve (linear range shown in Figure 4d). Thus, the calculated value of LOD for Ir-Au-Pt equals to ~0.12 µM and the maximum estimated sensitivity, which is proportional to the area of CV curve shown in Figure 4a, is ~9960 µA/mM cm^2^. The low detection limit and high sensitivity exhibited by Ir-Au-Pt can be explained by the large surface area of this material and the electrocatalytic synergy between iridium, gold and platinum. It is known that bimetallic and trimetallic Pt-based catalysts are desirable due to the presence of an additional metal that can enhance the catalytic activity via synergy because of the electronic, alloying or strain effects. In turn, all component of the alloy catalysts can affect the activity enhancement [33]. Moreover, not only the electrocatalytic activity, but also the stability of bimetallic and trimetallic noble metal nanocrystals (NCs) are higher than those of their monometallic counterparts due to the modification of their electronic structures, available catalytically active sites and other positive synergistic effects caused by structural and compositional differences between the monometallic and bimetallic NCs [34,35,36]. However, the mechanisms that lead to a synergistic effect and an increase of catalytic activity towards glucose electrooxidation are quite complex and individual for each composite; therefore, in order to clarify such mechanism for the material synthesized in this work the separate study is required. In addition, it is thought that active transition metal centers across the electrode and the presence of hydroxyl radicals play a crucial role in electrooxidation of glucose and many other organic molecules [24,37,38]. Therefore, taking into account the models known from literature and mentioned above, we can assume that catalytic oxidation of glucose on the surface of the synthesized Ir-Au-Pt microelectrode may proceed via the mechanism demonstrated in Figure 5.

**Figure 6:**
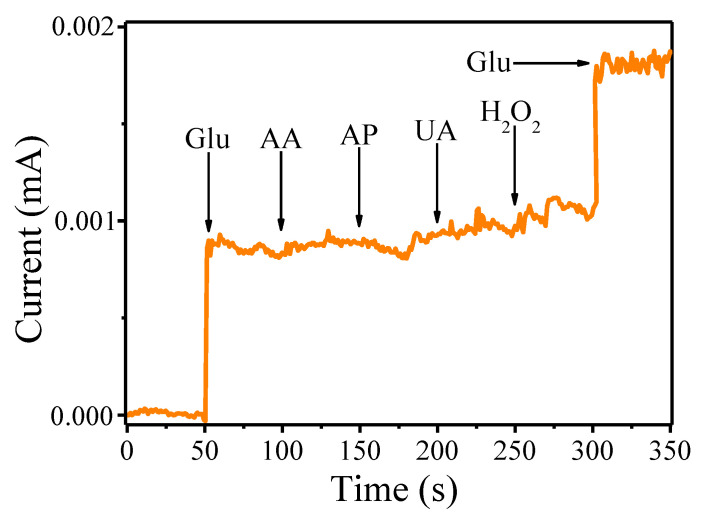
Amperometric response of Ir-Au-Pt microelectrode to successive addition of 50 μM D-glucose (Glu), 10 μM ascorbic acid (AA), 10 μM 4-acetamidophenol (AP), 10 μM uric acid (UA), 10 μM hydrogen peroxide (H_2_O_2_) in 0.1 M NaOH.

We have also tested the selectivity of the Ir-Au-Pt with respect to D-glucose in the presence of several interfering substances including ascorbic acid (AA), 4-acetamidophenol (AP), uric acid (UA) and hydrogen peroxide (H_2_O_2_) that typically coexist with glucose in the human blood (Figure 6). Figure 6 shows that addition of these substances to the background solution (0.1 M NaOH) leads to an increase of the amperometric current at the applied potential of 65 mV. The most prominent analytical response in comparison with other analytes was observed for D-glucose. Thus, according to these observations, it can be concluded that Ir-Au-Pt exhibits rather decent selectivity towards enzyme-free glucose sensing.

Furthermore, rather good electrochemical characteristics of iridium microelectrode modified with gold and platinum are accompanied with decent stability and reproducibility. Long-term stability was investigated with three Ir-Au-Pt microelectrodes for two weeks. As a result, it was observed that this material maintained ~91% its electrocatalytic activity with respect to non-enzymatic detection of D-glucose during the first week, whereas this activity dropped to ~85% during the second week. The great reproducibility was confirmed by low value of the relative standard deviation related to electrochemical response to 0.1 mM solution of D-glucose, which was found to be around 10% for three Ir-Au-Pt electrodes.

In summary, sensor characteristics of Ir-Au-Pt microelectrode were compared with same characteristics of non-enzymatic sensors based on similar metal and bimetallic micro- and nanostructures known from literature [7,27,28,39,40,41,42,43,44,45]. It should be noted that this microelectrode has a fairly low detection limit, a wide range of linearity and high sensitivity in relation to enzyme-free glucose sensing in comparison with many modern analogues (Table 2).

## 4. Conclusions

In this work, we used laser-induced metal deposition technique in order to manufacture several iridium-containing microelectrodes. It was found that modification of iridium electrode with gold and platinum both individually and as a mixture leads to better surface development and, as a result, enhances electrocatalytic activity towards non-enzymatic glucose sensing. In this regard, the most promising results were demonstrated by the composite microstructures based on iridium, gold and platinum exhibiting great electrochemical properties. It can also be noted that this material is of great interest for the design and development of inexpensive and effective portable sensor devices for enzyme-free determination of various biologically important analytes.

## Figures and Tables

**Table 1:** The compositions of solutions used for LCLD experiments.

Component	Concentration (mM)	Solvent
tris(2-phenylpyridine)iridium(III)	3	DMF
chloro(triphenylphosphine)gold(I)	3	DMF
dichloro(dicyclopentadienyl)platinum(II)	3	DMF

**Table 2:** Comparison of different electrode materials for non-enzymatic glucose sensing.

Electrode Material	Sensitivity (μA/mM × cm^2^)	Linear Range (mM)	Limit of Detection (μM)	Ref.
This work (Ir-Au-Pt)	9960	0.0005–1	0.12	-
IrO_2_ NFs Nafion/GCE	22.22	0–16	2.93	[28]
Ir-carbon	48.83	0–50	28,000	[39]
Pt-Ir	93.7	0–10	N/A	[27]
Nanoporous Pt	10.0	1–10	50	[40]
Pt NP	22.7	0–10	0.42	[44]
Pd-Cu-Pt	553	1–10	1.29	[41]
Pt-Au nanoporous film	12.85	0.2–4.8	1.3	[45]
Pt-nanoporous Au	145.7	0.5–10	0.6	[43]
Au nanocorals	22.6	0.0005–0.3	10	[7]
Au NP film	749.2	0.0556–13.89	9	[42]

## Supplementary Materials

The following are available online at https://www.mdpi.com/1996-1944/13/15/3359/s1, Figure S1: The experimental setup used for laser-induced deposition of iridium-containing microstructures: 1—cw 532 nm diode-pumped solid-state Nd:YAG laser, 2—mirrors, 3—splitting cube, 4—objective lens, 5—the experimental cell, 6—neutral density flter, 7—web camera, 8—personal computer, 9—the computer controlled motorized stage, Figure S2: EDX spectrum of iridium (I_r_) microstructures, Figure S3: EDX spectrum of iridium-platinum (Ir-Pt) microstructures, Figure S4: EDX spectrum of iridium-gold (Ir-Au) microstructures; Figure S5: XRD pattern of iridium (Ir) microstructures, Figure S6: XRD pattern of iridium-platinum (Ir-Pt) microstructures, Figure S7: XRD pattern of iridium-gold (Ir-Au) microstructures.
