# Supplementary material for: Laser-Induced Synthesis of Composite Materials Based on Iridium, Gold and Platinum for Non-Enzymatic Glucose Sensing
Source: Materials (Basel). 2020 Jul 29;13(15):3359. doi: 10.3390/ma13153359 (PMC7436056; doi:10.3390/ma13153359)
Supplement: Supplementary file 1 [file materials-13-03359-s001.pdf]

materials

Supplementary Materials

Laser-Induced Synthesis of Composite Materials Based on Iridium, Gold and Platinum for Non-Enzymatic Glucose Sensing

Maxim S. Panov [1], Evgeniia M. Khairullina [1], Filipp S. Vshivtcev [2], Mikhail N. Ryazantsev [1,2,*] and Ilya I. Tumkin [1,*]

[1] Saint Petersburg State University, 7/9 Universitetskaya nab., St. Petersburg 199034, Russia; m.s.panov@spbu.ru (M.S.P.); e.khayrullina@spbu.ru (E.M.K.)

[2] Nanotechnology Research and Education Centre of the Russian Academy of Sciences, Saint Petersburg Academic University, St. Petersburg 194021, Russia; vforvshivcev@gmail.com

* Correspondence: mikhail.n.ryazantsev@gmail.com (M.N.R.); i.i.tumkin@spbu.ru (I.I.T.)

Received: 23 June 2020; Accepted: 24 July 2020; Published: date

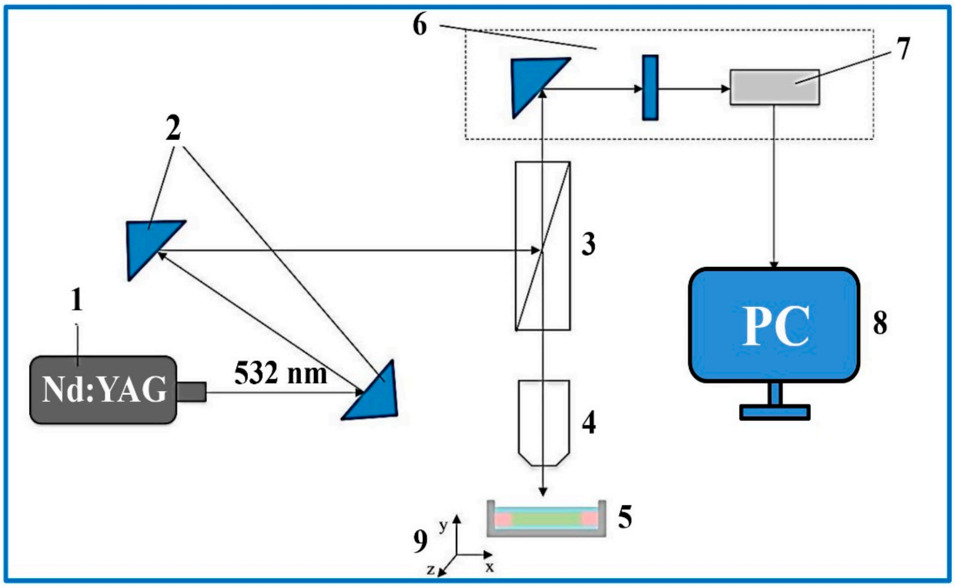

Figure S1. The experimental setup used for laser-induced deposition of iridium-containing microstructures: 1—cw 532 nm diode-pumped solid-state Nd:YAG laser, 2—mirrors, 3—splitting cube, 4—objective lens, 5—the experimental cell, 6—neutral density flter, 7—web camera,.

8—personal computer, 9—the computer controlled motorized stage.

The output from a continuous wave 532 nm diode-pumped solid-state Nd:YAG laser is split into two portions (Figure S1). First portion is focused between the plating solution and dielectric substrate, both of which are located in the experimental cell. The cell is translated horizontally and vertically by the computer controlled motorized stage in such a way that the focused laser beam is literally writing metal lines of almost any size and shape on the surface of a substrate. Second portion of the laser output travels to web-camera used for in situ monitoring the process of laser-induced metal deposition. Consequently, we were able to deposit the 8 mm long and 70–90 µm wide iridium (Ir) lines at the laser power of 1500 mW and at the scanning speed of 2.5 $\mu m \cdot s^{-1}$. Finally, the synthesized Ir lines were modified by the consecutive laser-induced deposition of gold and platinum on the top of them using the same deposition rate and at laser power of 1100 mW. Magnification and numerical aperture of the objective lens (Figure S1) were 50× and 0.95,

respectively. The laser spot size was about 4–5 µm and corresponding power density equals approximately to 2.1 mW µm^{-2}

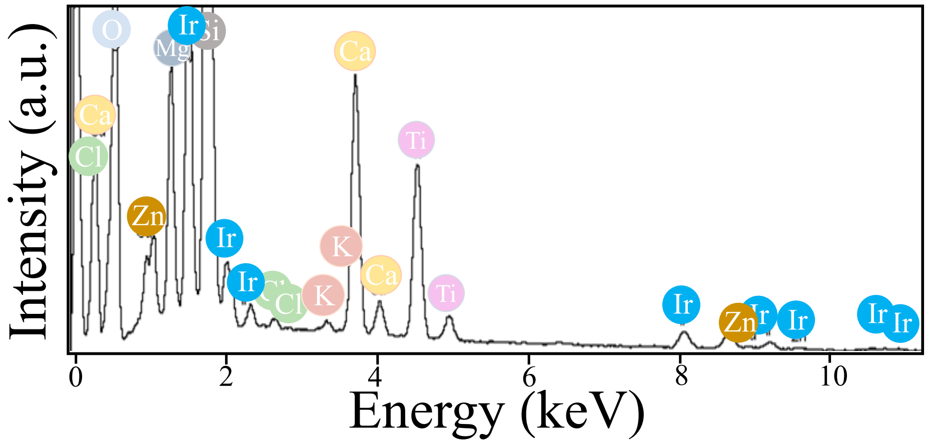

Figure S2. EDX spectrum of iridium (Ir) microstructures.

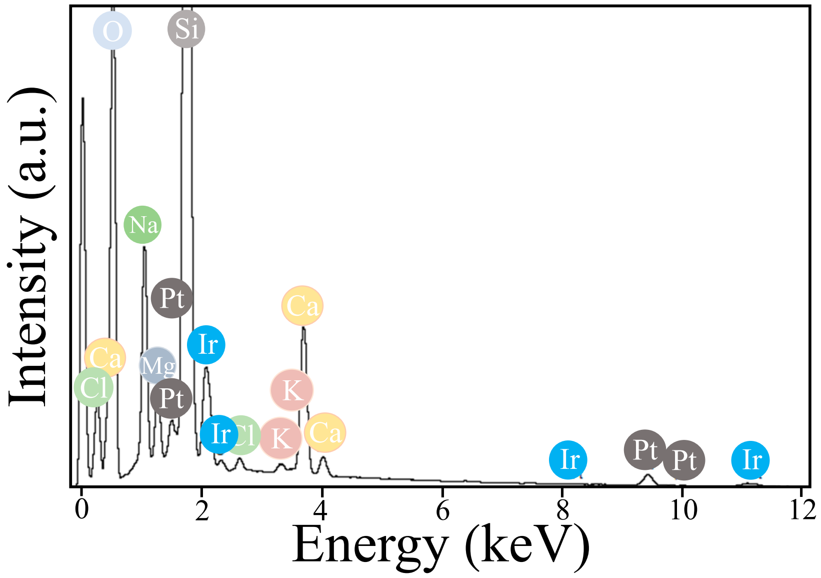

Figure S3. EDX spectrum of iridium-platinum (Ir-Pt) microstructures.

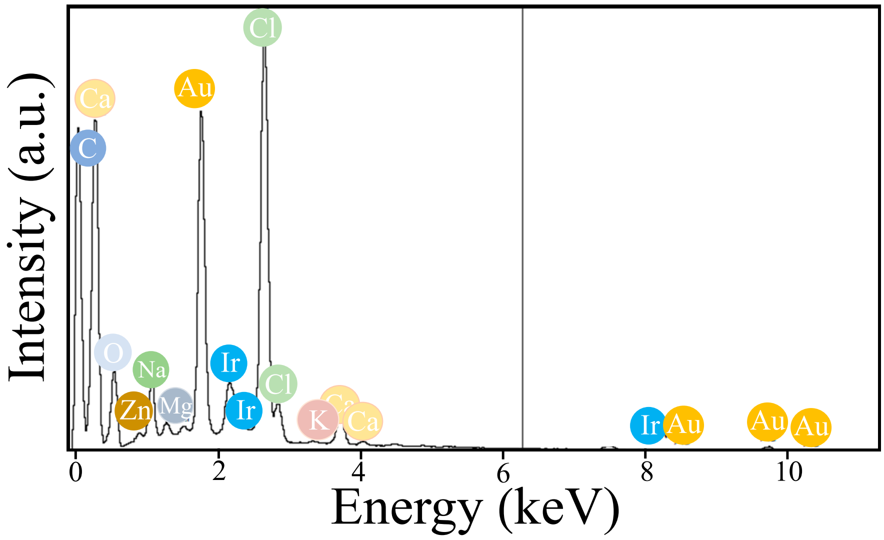

Figure S4. EDX spectrum of iridium-gold (Ir-Au) microstructures.

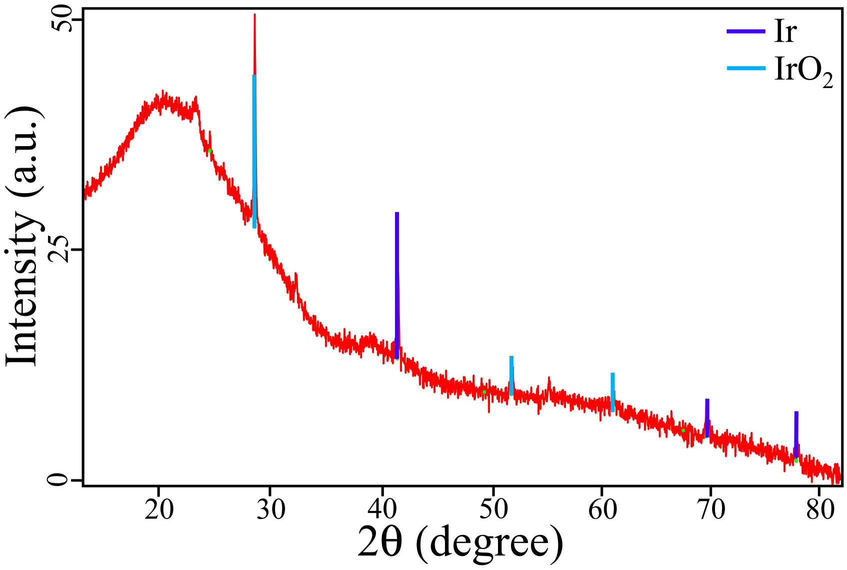

Figure S5. XRD pattern of iridium (Ir) microstructures.

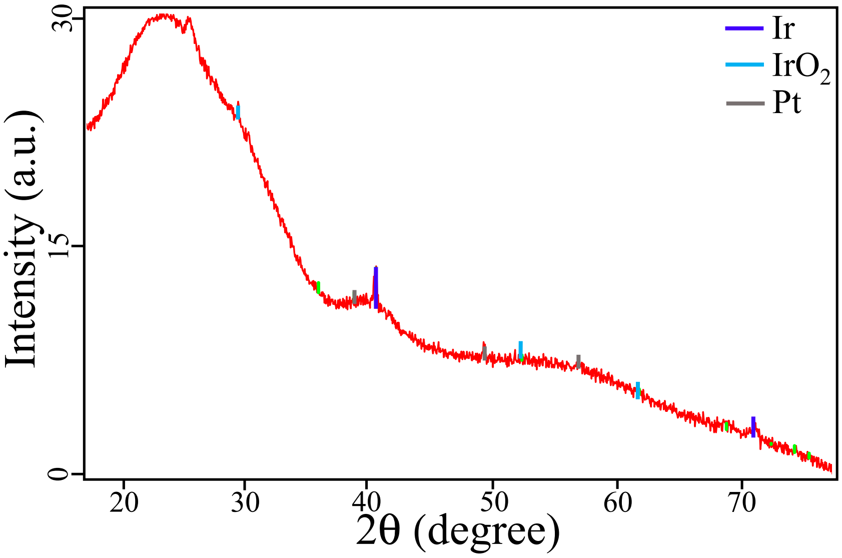

Figure S6. XRD pattern of iridium-platinum (Ir-Pt) microstructures.

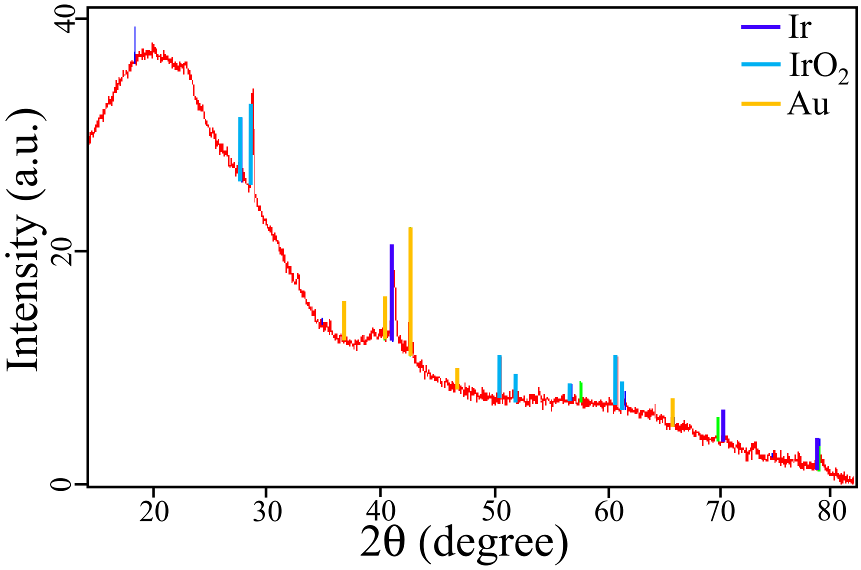

Figure S7. XRD pattern of iridium-gold (Ir-Au) microstructures.
